# Antecedents of the red-romance effect: Men’s attractiveness and women’s fertility

**DOI:** 10.1371/journal.pone.0284035

**Published:** 2023-04-11

**Authors:** Maria Agthe, Daniela Niesta Kayser, Sascha Schwarz, Jon K. Maner

**Affiliations:** 1 Department of Psychology, University of Potsdam, Potsdam, Brandenburg, Germany; 2 Department of Psychology, University of Hall in Tirol (UMIT Tirol), Hall in Tirol, Austria; 3 Department of Psychology, University of Berlin (FU Berlin), Berlin, Germany; 4 Department of Psychology, University of Wuppertal, Wuppertal, Nordrhein-Westfalen, Germany; 5 Department of Psychology, Florida State University, Tallahassee, Florida, United States of America; Chang-Qing Gao, Central South University, Xiang Ya Hospital, CHINA

## Abstract

The color red has been implicated in a variety of social processes, including those involving mating. While previous research suggests that women sometimes wear red strategically to increase their attractiveness, the replicability of this literature has been questioned. The current research is a reasonably powered conceptual replication designed to strengthen this literature by testing whether women are more inclined to display the color red 1) during fertile (as compared with less fertile) days of the menstrual cycle, and 2) when expecting to interact with an attractive man (as compared with a less attractive man and with a control condition). Analyses controlled for a number of theoretically relevant covariates (relationship status, age, the current weather). Only the latter hypothesis received mixed support (mainly among women on hormonal birth control), whereas results concerning the former hypothesis did not reach significance. Women (N = 281) displayed more red when expecting to interact with an attractive man; findings did not support the prediction that women would increase their display of red on fertile days of the cycle. Findings thus suggested only mixed replicability for the link between the color red and psychological processes involving romantic attraction. They also illustrate the importance of further investigating the boundary conditions of color effects on everyday social processes.

## Introduction

Color conveys a wealth of social information. The color red, in particular, has been implicated in a variety of social processes. Red appears to be relevant for a range of social contexts including power, dominance, anger, aggression [1, 2] and, most relevant to the current article, romantic attraction. For example, across a range of cultures, the color red is associated with love, lust, sexuality, and passion [3, 4]. Red was used to symbolize lust, passion, and fertility in ancient mythology and ritual [5–7], and red lipstick and rouge were used to enhance women’s appearance as early as 10,000 years ago in ancient Egypt [8]. Contemporary psychological research suggests that the color red is intimately associated with a range of mating-related perceptions and judgments [9]. At the same time, the existence of inconsistent and underpowered studies has generated legitimate questions about the robustness and replicability of the link between the role the color red and people’s mating-related psychology (e.g., [10–12]).

The current paper advances this literature by providing a reasonably powered conceptual replication and extension of work suggesting the link between the color red and interpersonal attraction. We build on previous research suggesting that women sometimes wear red because doing so increases their attractiveness to men [13]. Here we test two hypothesized antecedents of this effect. First, we assess whether women are more likely to display the color red when expecting to interact with a highly attractive man (compared with a less attractive man). Second, we assess whether women are more likely to display red when they are in the fertile (compared with less fertile) phase of their menstrual cycle. In the following sections, we describe previous evidence for women’s use of the color red, and present theoretical rationale for the examination of male attractiveness and female fertility as antecedents of women’s tendency to display the color red.

### The red-romance effect

The psychological association between red and romance can be seen throughout contemporary society, as illustrated in Valentine’s Day hearts and roses, in “red-light districts,” in the arts (literature, film, stage, song, e.g., “Lady in Red”), in fashion (lingerie, little red dress), and in the color of cosmetics such as lipstick and rouge. Empirical research suggests an attractiveness-enhancing effect of the color red both for men rating women (e.g., [13]) and for women rating men (e.g., [9]), although the evidence for the latter relationship is relatively weaker [14]. The color red seems to be associated with sexual attractiveness specifically [15], while black may increase people’s attractiveness more generally because it is seen as fashionable [16]. Empirical support for the “red-romance link” has been found using both self-report and behavioral indicators of attraction. For instance, compared to women dressed in other colors, women wearing red received higher attractiveness evaluations and were rated as more sexually desirable by men [13, 16, 17]. Such effects may reflect men’s interpretation of the color red as a signal of sexual interest [16]. Similarly, men asked women wearing a red shirt (compared to a blue or green shirt) more intimate questions and chose to place their seat closer to where a woman in red was expected to sit [18]. The red effect also applies to women displaying red on their lips or skin, as redness enhances the apparent femininity and attractiveness of women’s faces to men [19, 20]. This may have implications for people’s behavior. For instance, men were more willing to date and spend money on red-dressed women [13]. Although some recent replications of prior studies did not find that the color red enhanced positive responses (e.g., red did not enhance tipping behavior [21]), a recent meta-analysis documented the presence of small but reliable effects for both sexes, with women’s attractiveness being enhanced by the color red to about twice the degree as men’s attractiveness [14]. Yet, the meta-analysis also found evidence of publication bias, and pre-registered studies yielded smaller effects that could not always rule out null effects.

Nevertheless, several findings hint at the possibility that the association between red and sexuality is rooted in human evolutionary history such as the red-attraction-sexuality link in a rural society in Burkina Faso [22], hereby suggesting its cultural universality. Work also suggests that effects of the color red may be connected to perceptions of a woman’s underlying reproductive value [23]. The attractiveness-enhancing effect of the color red may reflect its link to perceived health, vitality, and sexual receptivity such as when women’s sexual arousal is accompanied by facial blushing [24–26], and when women’s facial skin becomes slightly redder during the fertile phase of their menstrual cycle [27, 28]. This body of research is consistent with the possibility that the link between red and attraction is rooted in human evolutionary biology. Through the repeated pairing of red and sexuality via cosmetics and lingerie, etc., cultural conditioning could, in turn, reinforce and extend this link [29, 30].

If women use the color red to enhance their attractiveness, they would be expected to do so more in some situations than others. Women’s mating preferences are not indiscriminate, and they are expected to enhance their attractiveness primarily in situations in which their mating goals are active and are interested in attracting a potential partner. In the current research, we test two contextual variables predicted to increase women’s display of the color red: (1) the presence of a highly attractive man and (2) being in the fertile period of the menstrual cycle.

### The role of male attractiveness

Women may be more likely to display the color red when expecting to interact with a man who is highly (versus less) attractive. Women tend to prefer as partners men who possess traits associated with judgments of physical attractiveness. Consistent with good genes theories of sexual selection [31–33], physical attractiveness represents one indicator of desirable traits such as health, dominance, and high genetic quality. Because women have benefited reproductively from mating with such men, the presence of a highly attractive man may activate women’s mating goals. Thus, if women display red as a sexual signal, they may do so more when interacting with a highly attractive (as compared with less attractive) man.

Some existing research is consistent with this hypothesis. For example, in one study, women were more likely to wear red over other-colored clothing when they expected to interact with an attractive man (versus an unattractive man or an attractive woman) [34]. In another study, women chose to wear red, and wore it more conspicuously when they expected to meet an attractive versus a less attractive man [35]. Additional research suggests that women’s preference for red attire emerges particularly in situations where the likelihood of encountering a potential mate is high (e.g., going to a party) versus where the chance of meeting a desirable partner is low (e.g., visiting one’s grandparents) [36]. Thus, we predicted that women would be more likely to display the color red when expecting to interact with an attractive man, compared with a less attractive man and compared with a control condition in which the attractiveness of the man was unknown. This hypothesis applies to naturally cycling women as well as to women taking hormonal contraceptives.

### The role of female fertility

A second hypothesized predictor of the red-romance effect involves the timing of a woman’s menstrual cycle. Research suggests that when women are in the fertile phase of their cycle–the period immediately prior to and through the time of ovulation–they experience heightened interest in mating. During their period of peak fertility, women feel more attractive [37–39], feel more sexual desire, and report heightened interest in attending social gatherings where they might actually meet a prospective mate [38]. Around the time of peak fertility, women also tend to wear more provocative [39] and more revealing clothing [40–42]. These behaviors are consistent with women’s desire to impress men by enhancing their attractiveness [42] and their desire to outshine potential same-sex rivals [40].

If displaying the color red serves as a mating tactic, women would be expected to display that color especially when they are in the fertile phase of their menstrual cycle. Indeed, some existing evidence supports this hypothesis. For example, some research indicates that women buy and use more appearance-enhancing styling and attention-grabbing products, such as red lipstick or red-colored clothing [38, 43–45]. Women also are more likely to choose red (and also pink) attire during the fertile phase [46–48]. Eisenbruch and colleagues [47] suggested that the hormonal “estradiol-to-progesterone ratio” mediates this fertility effect in that it predicted the odds of wearing red clothing on fecund (versus infertile) cycle days. In line with such findings, we predicted that when women are in the fertile phase of their cycle, as compared with other times during their cycle, they would be more likely to display the color red. The group of women who take hormonal contraceptives is considered a control group for this hypothesis. That is, they are not expected to show the effect of cycle phase on red display that is predicted for naturally cycling women (i.e., more display of red at peak fertility).

### The current research

The field has generated several pieces of intriguing evidence supporting the link between the color red and mating-related psychology. Nevertheless, the replicability of these findings has been questioned [10, 11]. The current research sought to conceptually replicate and extend the body of evidence testing hypothesized antecedents of the red-romance effect. We generated a reasonably powered test of whether women would display red to a greater extent when 1) they anticipated interacting with a highly attractive man (compared with a less attractive man and a control condition), 2) they were in the fertile (compared with less fertile) period of their menstrual cycle. We assessed both whether or not women wore red (dichotomous variable) and the degree to which they wore red (continuous variable).

In addition to testing for these effects, we measured a number of theoretically relevant covariates. First, we assessed participants’ relationship status. Single women might be expected to experience greater interest in meeting potential new partners, and so might be expected to use red strategically as a mating tactic. Therefore, we accounted for whether women were single versus in a committed relationship. Second, we assessed participants’ age. Because a person’s age can affect the desired age of potential partners [49], we accounted for potential effects associated with women’s age. Third, we assessed the current weather conditions. Women might use the color red as a mating tactic more when the weather is cold or dreary, as compared to when it is sunny, when women might display romantic interest by wearing less clothing [48; see also 41; 46]. Whether women’s effects of fertility status might depend on the current weather has not been investigated, we additionally conducted analyses with and without these covariates to evaluate the robustness of any observed effects.

As a conceptual replication, the current study’s procedure largely corresponds to a prior study of Niesta Kayser, Agthe, and Maner [35], which indicated that women chose to display the color red when expecting to interact with an opposite-sex experimenter. The current work parallels that study in its use of the same stimulus pilot testing, experimental protocol, participant compensation, coding procedure of the display of red, a priori exclusion criteria, and covariates). Yet, our current study was a conceptual replication and not a direct replication, as we used three experimental groups (attractive picture vs. average-looking picture vs. no picture), whereas Niesta Kayser et al. [35] only included two experimental conditions (highly attractive vs. less attractive picture of the male experimenter) and compared them to a baseline consisting of a large sample of women in a natural environment with no induced expectation. Aside from using a more rigorous control group, we included an additional independent variable (high vs. low fertility) and an additional covariate (weather) to also attend to prior findings on the effects of fertility and weather on women’s display of the color red (e.g. [46–48]). As prior research had shown effects of fertility on women’s choice of wearing red or pink [46], but had not included some possibly important covariates, we had a priori plans to include three covariates (age, weather, and relationship status). Moreover, to amass sufficient statistical power, we used a larger sample size than Niesta Kayser et al. [35]. Thus, our current conceptual replication parallels prior research, but cannot be considered a direct replication.

## Materials and methods

### Participants

We conducted power analyses to ensure a sufficiently sized sample. Sample size calculation for logistic regression (for the dichotomous dependent variable: participant wore red or not) following the work of Peduzzi and colleagues [50] provides the guideline for a minimum number of cases based on the number and proportion of cases with a negative (Y = 0) and positive (Y = 1) outcome. We estimated the baseline proportion of positive cases (p) in the population (22.2% of women displaying red in the less attractive condition in Niesta Kayser et al. [35], and the number of independent variables (k) was set to two (i.e., experimenter attractiveness and fertility). This calculation resulted in a sample size of N = 10k / p, yielding a minimum number of cases of N = 135 to achieve a power of .90. We wanted to ensure adequate power, so we recruited well over twice that number of participants (N = 281). For the continuous dependent variable (degree of red), we conducted an a priori power analysis based on an estimated effect size of 0.57 (Cohen’s *d*s), derived from a previous study investigating the strategic sexual signalling effect [35]. That analysis suggested that a total sample of *N* = 150 participants was sufficient to achieve a power of .90.

Participants were 281 women younger than 50 years of age (range: 18–46 years). All were pre-menopausal and none was pregnant at the time of the experiment. Overall, a total of 323 participants participated in this experiment, but based on a priori exclusion criteria we excluded two participants who were older than 50 years old and self-reported to be menopausal, three participants who reported being pregnant, one participant who refused to have her photo taken, and one participant who failed to complete the questionnaire. Moreover, 11 participants who reported being lesbian were excluded from the analyses, because we only manipulated the physical attractiveness of a male research assistant and thus our hypotheses were limited to heterosexual women. Further, a total of 20 participants did not report the necessary data to calculate the fertile window, so they were excluded, as well.

We made the decision a priori to limit analyses to European Caucasian participants. Previous findings document not only biases in how people judge the physical attractiveness of other-race faces (e.g., [51]), but also preferences for members of one’s own race as potential mates [52, 53]; this tendency may be especially pronounced for White participants [54, 55]. The ostensible interaction partner (a research assistant) was White, and thus we focused analyses on European Caucasian participants. To avoid discrimination, however, all women who signed up for the study were allowed to take part regardless of their ethnicity. Analyses including all participants are presented in S1 File. In sum, data from 281 heterosexual and bisexual participants between 17 and 46 years (*M* = 21.72, *SD* = 4.78) were used in primary analyses (primarily psychology students: 70.11%). Data from the complete sample of 285 women (*M* = 21.74, *SD* = 4.77; primarily psychology students: 69.48%) are reported in the S1 File. Participants were each compensated 5 Euros for taking part in the study.

### Approval of the ethics committee and documentation of a priori predictions

This study was approved by the Ethics Committee of the Department of Psychology and Education of the Ludwig-Maximilians-University (Number: DFG Ni/1115/2-1) in Munich (Germany) as well as by the Ethics Committee of the Department of Psychology of the University of Wuppertal (Germany) (Number: DFG Ni/1115/2-1; part labelled Schw 1511.3–1 of the DFG project) in accordance with the ethical standards expressed in the Declaration of Helsinki, and its approval was first documented already in 2013. The approved DFG grant contained the main a priori predictions of our current study (i.e., study 4b of DFG Ni/1115/2-1). Its peer-reviewed laboratory protocol was the same as in the previously published study which it tried to replicate [35] (study 4a of DFG Ni/1115/2-1), but it was extended by a more rigorous control group. The current study was conducted after the publication of Niesta Kayser et al. (2016) [35]. It used the same material (e.g., pictures of stimulus persons), the same exclusion criteria, and the same main predictions, as the article it sought to replicate [33]. All participants gave informed consent and were thoroughly debriefed. The individuals’ consent was obtained at two separate points: first it was obtained after individuals responded to the contact email by affirming that they wanted to participate (this affirmative expression was qualified written consent). The second (verbal) consent was obtained before and after the participants had their photograph taken. They were thoroughly informed that they did not need to participate in this additional procedure and assured that they could discontinue and leave the experiment at any point of time. When the experimenter asked for the participant’s consent, he emphasized that they would receive their credit even if they decided not to participate in this study. Verbal consent was considered to be sufficient, because it was ensured that data were stored and analysed anonymously. We also considered verbal consent to be more appropriate (compared with written consent): Obtaining verbal consent guaranteed the participants that neither their name nor their signature was associated with their picture in any way, hence guaranteeing the participants’ anonymity. Additionally, both before and after taking the picture the participants were reassured that they could discontinue the study at any point. No one withdrew consent to having their picture used in this study and all participants signed a written consent at the end along with a receipt of the participation reward. The photographs are used solely for data analysis and are not published. The procedure of verbal informed consent including the consent protocol was approved by the Ethics Committee based on this information.

### Design and procedure

To vary the attractiveness of an anticipated interaction partner, we varied the attractiveness of the research assistant who ostensibly would be conducting the session. Each participant received an instruction email two days prior to the day of her session, and that email included a photo of the alleged research assistant. Participants were assigned to either an attractive or an average-looking male experimenter condition. The attractiveness of seven male facial photos was rated in a pilot study with 20 female students using a 9-point scale. Based on these attractiveness ratings, we chose an above average male image (*M* = 6.63, *SD* = 0.89) for the high attractiveness condition (*n* = 101) and an average-looking male image (*M* = 3.90, *SD* = 1.35) for the lower attractiveness condition (*n* = 108). A third group of participants (control group, *n* = 72) received an identical reminder email, which did not display a photo of the experimenter. Unfortunately, although all participants were randomly assigned to the experimental conditions by the research assistant, the number of participants in the control group was somewhat lower than the number of the other two conditions due to experimenter error. Due to miscommunication among the research team, the research assistant at the Ludwig-Maximilians-University of Munich initially omitted the no-picture control condition from data collection. Although we realized and corrected this error quickly (so that there was a randomization between three instead of two conditions; attractive target vs. less attractive target vs. no picture included), there were fewer participants in the control condition at the end of the semester when the study ended. To achieve a sufficient number of participants, the study was conducted at the Ludwig-Maximilians-University of Munich and at the University of Wuppertal at the same time, following the same protocol as the study being replicated (which had been approved by the ethics committee of the respective university as delineated above). The research assistants who conducted the study (one in Munich and one in Wuppertal) did not know the hypotheses of the study, they were not involved in any research on effects of the color red (one of them has a congenital color blindness with regard to red and green), and they were not involved in any way in the color coding (the color coding was conducted by research assistants of the University of Potsdam as delineated below in the separate paragraph regarding color coding).

Upon arriving for the session, participants in the two attractiveness conditions were informed that the experimenter displayed in the reminder email could not attend the session. Instead, the study was run by a different experimenter. To disguise the study’s purpose, the participant then completed another, ostensibly unrelated study titled “intelligence and perception.” After the participant finished the study, she was asked if she would agree to have a photo taken of her (ostensibly for a different study) and was offered an incentive of 5 Euros (above the 5 Euros they earned for participation). All but one participant agreed to this request.

The experimenter took two photos of each participant, a close-up of her face and a photo of her entire body, to ensure that all aspects of her clothing, accessories, and makeup were visible. We kept constant the conditions in which the photos were taken (e.g., the amount of the light influence was equivalent; the location was always in front of the same off-white wall in the lab). After the photographs were taken, participants reported demographic information and completed a questionnaire containing information about their menstrual cycle (details below), and relationship status (details below). Participants were then probed for suspicion and debriefed. Three participants indicated that they were suspicious when they received the experimenter’s reminder email containing the photo. Results were identical whether or not those three participants were retained in the analyses; thus, the results reported below include those participants.

Data were analyzed using JASP [56]. Our data is available by the following link: https://osf.io/q47rj/

#### Estimation of fertility

Participants reported menstrual cycle information adopted from questionnaires used in previous research to estimate timing of fertility [42, 57, 58]. Participants also reported on hormonal contraception use. We estimated fertility in normally ovulating women (*n* = 152) by first estimating the onset of the next menstrual cycle given their reported average cycle length, and the first day of the last onset of the menstrual cycle. Then, we estimated the day of ovulation 15 days prior to the onset of the next menstrual cycle. Finally, consistent with recommendations by Gangestad et al. [59], we categorized women to be in the high-fertile phase of the cycle if they attended the lab at the day of ovulation or up to 8 days preceding the day of ovulation (*n* = 39). Otherwise, they were estimated to be in the low-fertile phase of the cycle (*n* = 113). Nine participants did not provide sufficient data to estimate fertility.

An additional 129 women were using hormonal contraceptives and served as a control group for analyses involving menstrual cycle phase. Although we classified the timing of their participation as “high-fertile” (*n* = 39) or “low-fertile” (*n* = 90) phase of the cycle, those women do not experience the same hormonal fluctuations as normally cycling women do. Thus, if changes in the use of the color red are driven in part by hormonal changes characteristic of high fertility, we would expect increases in the use of red in the highly fertile phase by normally cycling women, but not by women on hormonal contraceptives.

#### Color coding

To code participants’ appearance, we used procedures described in previous research [46] according to the same protocol that had already been approved by the according ethics committee (please see above). The range of reddish hues included pink, red and scarlet, thereby including prototypical shades of red, but excluding atypical shades of red, such as orange, maroon or purple. Two independent research assistants at the University of Potsdam (who had no contact to the research assistants in Munich and in Wuppertal who had conducted the study), completely blind to hypotheses and experimental condition, coded use of the color red in participants’ clothes, accessories, and make-up. Before coding the pictures, they received the part of the laboratory protocol on how to do the color coding. They were instructed by the leading researcher at the University of Potsdam and received a short training on unrelated pictures. In coding the color of the participant photographs, the independent raters used the same, calibrated monitor/display in the research lab. Female participants were categorized as displaying red if they showed red on any part of their clothing (e.g., shirt or dress), accessories (e.g., bag or scarf) or make-up (e.g., lipstick). Inter-coder agreement was good (Kappa > .90 *p* < .001); a third rater (another research assistant at the University of Potsdam) resolved discrepancies. In addition to this dichotomous measure, we generated two continuous measures based on how much red each participant showed and how obviously red was displayed, each using a 5-point scale from 0 (not at all) to 5 (very much). These two measures were correlated (*r* = .915, *p* < .001) and were averaged to form a composite measure.

#### Current weather

Weather was coded by the respective research assistant at the Ludwig-Maximilians-University of Munich and at the University of Wuppertal who conducted the study and took the pictures, because the weather of the exact day of participation of each woman taking part in the study needed to be documented to be able to control for possibly influences of the weather. The research assistants were unaware of the hypotheses. They coded the weather as “sunny” (*n* = 84), “rainy / snowy” (*n* = 72) and “mixed / cloudy” (*n* = 125). These data were recoded to “good weather” (*n* = 84) and “bad weather” (*n* = 197).

#### Relationship status

To assess women’s relationship status, we coded women as single (*n* = 133, coded 0: “I’m single and dating someone” (*n* = 68) or “I’m single and not dating someone” (*n* = 65)) or in a committed relationship (*n* = 148, coded 1: “married” (*n* = 11) or “I’m in a close relationship” (*n* = 137)).

All conditions of the current study are reported in this manuscript.

## Results

We evaluated whether women’s display of the color red would be (1) affected by the attractiveness of the male experimenter and (2) associated with women’s level of fertility. Both of those predictors were included simultaneously in each model. In addition, to evaluate the robustness of each model, we repeated each analysis including participant age, relationship status, and the current weather as covariates.

Descriptive data are provided below in Table 1.

**Table 1:** Red display of women in the attractive and less attractive experimenter and control condition.

Stimulus attractiveness	Red not displayed	Red displayed	Total
Attractive	49	52	101
	48.5%	51.5%	100%
Control	48^a^	24^b^	72
	66.7%	33.3%	100%
Less attractive	79^a^	29^b^	108
	73.1%	26.9%	100%
Total	176	105	281

*Note. Significant (p < .05) between-column differences are marked by different superscripts.

### Normally cycling participants

**Fig 1:**
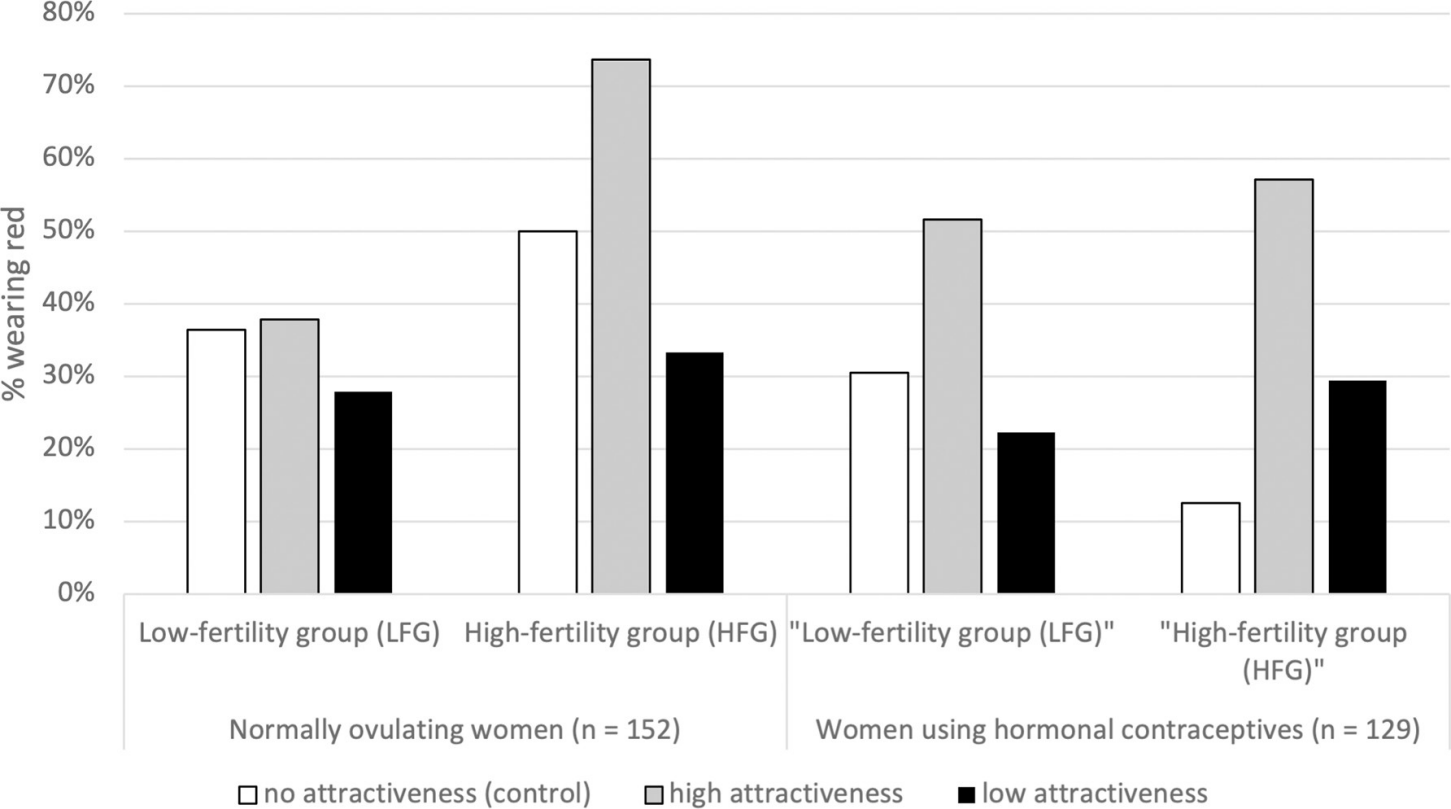
The effects of the physical attractiveness of the male research assistant and “fertility” in normally ovulating women versus women taking hormonal contraceptives.

We first focused on normally cycling women. When analyzing the dichotomous measure (wore red or not), as can be seen in Fig 1 (left panel), 73.7% of the participants in the high-fertile phase of the cycle expecting an attractive research assistant wore red compared to 37.8% participants in the low-fertile phase of the cycle awaiting the attractive research assistant, *Chi*^*2*^(1) = 6.45, *p* = .011, *n* = 56), proportion difference 35.84%, 95% CI = [0.11, 0.61]. Comparable effects were not identified for women in the control condition (who did not expect an attractive or less attractive research assistant), *Chi*^*2*^(1) = 0.50, *p* = .48, *n* = 41, proportion difference 13.64%, 95% CI = [-0.25, 0.52]) or the less attractive research assistant, *Chi*^*2*^(1) = 0.13, *p* = .71, *n* = 55), proportion difference 5.42%, 95% CI = [-0.24, 0.35].

To be able to compare our results to prior research (which had shown effects of fertility on women’s choice of wearing red or pink [46], but which had not included some possibly important covariates), we included *Chi*^*2-*^analyses, but as we had a priori plans to include three covariates (age, weather, and relationship status), we further tested this effect with a logistic regression analysis predicting the odds of wearing red from fertility (low-fertile vs. high-fertile), attractiveness of the research assistant (dummy coded to compare each condition to control), and age, relationship status, and weather as covariates. This analysis showed an OR = 2.74 for the attractive research assistant x fertility interaction, which was not statistically significant in this full model (z = 0.98, p = .33) (see Table 2).

**Table 2:** Regressing the likelihood of wearing red (0: no vs 1: yes) from the attractiveness of the research assistant, fertility, attractiveness x fertility interaction, controlling for age, relationship status and weather for normally cycling participants.

						Wald Test		
Predictor	Estimate	Robust SE	OR	95% CI OR	z	Wald Statistic	df	p
Attractive RA (vs control) (AR)	0.01	0.50	1.01	[0.38, 2.69]	0.03	< 0.001	1	.98
Unattractive RA (vs control) (UR)	-0.34	0.51	0.71	[0.26, 1.93]	-0.66	0.44	1	.51
Fertility (F)	0.56	0.78	1.75	[0.38, 8.09]	0.71	0.48	1	.48
AR x F	1.01	1.03	2.74	[0.37, 20.45]	0.98	0.97	1	.33
UR x F	-0.52	1.06	0.60	[0.07, 4.79]	-0.49	0.22	1	.63
Age	-0.03	0.05	0.97	[0.89, 1.06]	-0.61	0.48	1	.54
Relationship status	-0.11	0.42	0.89	[0.39, 2.05]	-0.27	0.08	1	.79
Weather	0.69	0.49	1.99	[0.76, 5.26]	2.13	0.16	1	.16

Notes. *N* = 152 normally cycling participants; SE = Standard error, OR = Odds Ratio, CI = Confidence Interval, RA = Research assistant, Attractive RA (AR) was coded as attractive RA (1) vs control group (0), Unattractive RA (UR) was coded as unattractive RA (1) vs control group (0), Fertility (F) was coded as 0 (low) vs 1 (high) estimated fertility, Relationship status was coded as not in a committed relationship (0) vs in a committed relationship (1), Weather was coded as bad weather (0) vs good weather (1)

The continuous dependent measure, that is the composite of the two z-transformed variables “how much” and “how obvious” red was used in the attire, was influenced by the attractiveness of the research assistant (F(2, 146) = 3.628, p = .029, partial Eta2 = .045). Post-hoc simple contrast analysis revealed that participants wore significantly more red in their attire when awaiting an attractive research assistant (M = 0.37 [95% CI 0.11, 0.64]) compared to the control condition (M = -0.14 [95% CI -0.51, 0.23], t(146) = 2.25, p = .026, d = .55. However, women awaiting the less attractive research assistant (M = -0.078 [95% CI -0.38, 0.23] did not differ in their display of red from the control condition (t(146) = 0.26, p = .795, d = .07). Further, women in the HFP (M = 0.24 [95% CI -0.077, 0.56]) wore more and more obvious red than women in the LFP (M = -0.14 [95% CI -0.31, 0.04]), F(1, 146) = 4.194, p = .042, partial Eta2 = .028). The attractiveness x fertility interaction was not significant (F(2, 146) = 1.858, p = .16, partial Eta2 = .025). An ANCOVA including age, relationship status and weather as covariates showed, that the main effects of experimenter attractiveness (F(2, 143) = 2.878, p = .06, partial Eta2 = .039) and fertility (F(1, 143) = 3.697, p = .056, partial Eta2 = .025) were not significant. The attractiveness x fertility interaction is also not significant in this set of analysis (F(2, 143) = 2.297, p = .104, partial Eta2 = .031) (see Table 3). Please note that because the analyses use the mean scores of the two items that are z-transformed, some of the values are lower than zero.

**Table 3:** The effects of the attractiveness of the research assistant, fertility, research assistant x fertility interaction, controlling for age, relationship status, and weather.

Variable	Sum of squares	df	Mean square	F	p	η^2^_p_
Attractiveness of the RA (RA)	5.04	2	2.52	2.88	.06	0.04
Fertility (F)	3.24	1	3.24	3.70	.056	0.03
RA x F	4.02	2	2.01	2.30	.10	0.03
Age	2.08	1	2.08	2.38	.13	0.02
Relationship status	1.38	1	1.38	1.58	.21	0.01
Weather	1.21	1	1.21	1.39	.24	0.01
Residuals	125.17	143	0.88			

Notes. *N* = 152 normally cycling participants, Fertility (F) was coded as 0 (low) vs 1 (high) estimated fertility, Relationship status was coded as not in a committed relationship (0) vs in a committed relationship (1), Weather was coded as bad weather (0) vs good weather (1)

### Hormonal contraceptive users

The same set of analyses was conducted on women using hormonal contraceptives. As can be seen in Fig 1 (right panel), no effects of “fertility” were observed in these women. Again, we conducted a logistic regression analysis predicting the odds of wearing red from “fertility” (“low-fertile” vs. “high-fertile”), attractiveness of the research assistant, and age, relationship status, and weather as covariates. Although the odds of wearing red was 2.17 times higher in good compared to bad weather (see Table 4), this model did not reveal a significant effect of weather (*p* = .07).

**Table 4:** Regressing the likelihood of wearing red (0: no vs 1: yes) from the attractiveness of the research assistant, fertility, attractiveness x fertility interaction, controlling for age, relationship status and weather for hormonal contraceptive users.

						Wald Test		
Predictor	Estimate	Robust SE	OR	95% CI OR	z	Wald Statistic	df	p
Attractive RA (vs control) (AR)	0.70	0.60	2.01	[0.61, 6.54]	1.15	1.35	1	.25
Unattractive RA (vs control) (UR)	-0.55	0.65	0.58	[0.16, 2.08]	-0.84	0.77	1	.40
“Fertility” (“F”)	-1.08	1.08	0.34	[0.04, 2.83]	-1.00	0.84	1	.32
AR x “F”	1.45	1.24	4.26	[0.37, 48.76]	1.16	1.16	1	.24
UR x “F”	1.57	1.28	4.81	[0.39, 59.27]	1.23	1.33	1	.22
Age	0.009	0.04	1.01	[0.93, 1.10]	0.22	0.04	1	.83
Relationship status	0.36	0.47	1.43	[0.57, 3.59]	0.76	0.63	1	.45
Weather	0.77	0.43	2.17	[0.93, 5.03]	1.80	3.38	1	.07

Notes. *N* = 129 hormonal contraceptive users; SE = Standard error, OR = Odds Ratio, CI = Confidence Interval, RA = Research assistant, Attractive RA (AR) was coded as attractive RA (1) vs control group (0), Unattractive RA (UR) was coded as unattractive RA (1) vs control group (0), “Fertility” (F) was coded as 0 (low) vs 1 (high) estimated fertility similar to the normally cycling group, Relationship status was coded as not in a committed relationship (0) vs in a committed relationship (1), Weather was coded as bad weather (0) vs good weather (1)

However, the attractiveness of the research assistant significantly affected the continuous composite variable (i.e., quantity and obviousness) of red women displayed, F(2, 123) = 4.75, *p* = .01). Post-hoc tests showed that women using hormonal contraceptives display significantly more red when awaiting an attractive research assistant compared to the control condition, t(123) = 2.70, p = .008, *d* = .64), and significant more red compared to the low attractiveness condition, t(123) = 2.56, *p* = .01, *d* = .52). No significant differences were found between the control and the unattractive research assistant condition, t(123) = 0.58, p = .56). No further significant effects were found in the main analysis (Fs < 1). An ANCOVA including age, relationship status and weather as covariates showed that the main effect of attractiveness is still significant in this model, F(2, 120) = 3.60, p = .03). However, weather is no significant covariate in this model, F(1, 120) = 3.80, p = .054). There were no other significant effects (Fs < 2.34, p > .104) (see Table 5).

**Table 5:** The effects of the attractiveness of the research assistant, “fertility”, research assistant x “fertility” interaction, controlling for age, relationship status, and weather.

Variable	Sum of squares	df	Mean square	F	p	η^2^_p_
Attractiveness of the RA (RA)	6.28	2	3.14	3.60	.03	.06
Fertility (“F”)	0.10	1	0.10	0.11	.74	< .001
RA x “F”	0.85	2	0.43	0.49	.62	0.008
Age	1.14	1	1.14	1.30	.26	0.01
Relationship status	2.34	1	2.34	2.68	.10	0.02
Weather	3.32	1	3.32	3.80	.054	0.03
Residuals	104.79	120	0.87			

Notes. *N* = 129 hormonal contraceptive users; “Fertility (F)” was coded as 0 (low) vs 1 (high) estimated fertility similar to normally cycling participants, Relationship status was coded as not in a committed relationship (0) vs in a committed relationship (1), Weather was coded as bad weather (0) vs good weather (1)

### Conceptual replication: Comparison of the current study’s results with prior research

Our conceptual replication mainly refers to the recent study of Niesta Kayser et al. [35]. We conceptually replicated the finding that women displayed more red when expecting to interact with an attractive man (compared to women expecting a less attractive interaction partner). Yet, as our current study (1) used three experimental groups (attractive picture vs. average-looking picture vs. no picture), whereas Niesta Kayser et al. [35] had compared the experimental conditions (highly attractive vs. less attractive picture) to a baseline (consisting of women in a natural environment with no induced expectation), and as (2) we additionally investigated whether women’s display of the color red would be associated with women’s level of fertility, our analyses could not exactly parallel the analyses of Niesta Kayser et al. [35]. That is, in our current model, both predictors (i.e. stimulus attractiveness and women’s fertility) were included simultaneously in each model, whereas Niesta Kayser et al. [35] used logistic (binary) regression with attractiveness condition as the predictor variable. Irrespective of these differences in analyses, the finding that women’s display of the color red was influenced by the attractiveness of an expected male interaction partner was replicated, but unexpectedly it only reached significance for women using hormonal contraceptives.

## Discussion

Although the color red has been implicated in a variety of social processes, the replicability of this literature has been questioned. The current research conducted a reasonably powered conceptual replication to investigate (a) whether women are more likely to wear red (and wear quantitatively more red) during the fertile window than on other cycle days and (b) whether women show a higher likelihood of wearing red (and wear quantitatively more red) when expecting to interact with an attractive (versus less attractive) man. Altogether, when rigorously testing our hypotheses, analyses revealed that despite support in a simple model, correction for important covariates reveals that only one of our six of the predictions was supported. The current results cannot confirm the hypothesis that women use the color red strategically when likelihood of conception is highest. In addition, the effect of experimenter attractiveness was significant in women on birth control (p = .03) but only trended toward significance in normally cycling women (p = .06). Thus, we saw mixed support for the prediction that women would wear more red in response to an attractive male interaction partner.

Although prior findings indicate that self-promotion appears to be a common strategy to compete for potential mates and to enhance one’s reproductive success (e.g., [60, 61]; for an overview see [62]), our current results were less conclusive. Thus, although prior studies documented links between the enhancement of attractiveness and fertility, it might be possible that the role the color red plays in attraction is not as strong as previously claimed.

Only women taking hormonal contraceptives, but not naturally cycling women, showed significantly more red in their outfit when expecting to interact with an attractive research experimenter (compared to a less attractive experimenter or compared to the control condition where no picture of the alleged experimenter was included). Our prediction that women would be more likely to show the color red in their clothing when they expected to interact with an attractive experimenter was not fully confirmed, as results for the naturally cycling women did not quite reach conventional levels of statistical significance when controlling for important covariates (age, relationship status, and weather).

It might be worthwhile for future research to further investigate whether naturally cycling women might use other ways of enhancing their attractiveness (e.g., more revealing clothing, more fashionable clothes) than relying on the color red. It might also be interesting to test whether or not naturally cycling women might, at the peak of fertility, have a high threshold for finding a potential interaction partner attractive, which would presumably result in signs of attraction only when presented with a target who was especially high in attractiveness. Nonetheless, in the current study there was no significant effect of experimenter attractiveness or fertility on red display for naturally cycling women, when controlling for age, relationship status, and weather.

Regarding investigations of the color red on mating-related behavior, one strength of our current study is that we measured women’s natural behavior (i.e., spontaneous choice of their outfit from their own clothing, accessories, and make-up instead of a forced choice from a range of presented clothing and attire) in their natural environment. Moreover, the study included experimental conditions involving not only a highly attractive and less attractive man, but also a true baseline control condition. This allowed us to ascertain whether expecting to interact with an attractive man increases women’s use of red, whether expecting to interact with a less attractive men decreases the use of red, or some combination of the two. In the current study, we found stronger evidence for the former evidence than for the latter. While women (taking hormonal contraceptives) displayed more red when expecting to interact with a highly attractive man, women expecting to interact with a relatively less attractive man did not decrease their use of red (compared with control). It should be noted, however, that the less attractive man in the current study was not particularly unattractive. It remains for future research to assess whether a highly unattractive man might reduce the extent to which women display the color red, and perhaps other signs of romantic attraction.

In the current study, we were able to compare findings for women using versus not using hormonal contraceptives. As expected, we saw no menstrual cycle effects among women on hormonal contraceptives. Yet, we saw no effects of fertility among naturally cycling women either. Thus, as mentioned before, more research is needed to assess whether and when the display of red is enhanced for women in their fertile phase.

No significant effects of current weather on displays of red were observed. It remains for future research to investigate more rigorously whether the use of color might be affected by the weather (e.g., whether sunshine may indirectly, possibly via hormonal changes and according changes in motivation, mood and potentially self-assurance, enhance the display of red as a means of appearing more attractive). We report results regarding the weather in more detail in our S1 File.

### Limitations and future research

One methodological limitation of the present research was our reliance on self-report rather than hormonal measures to assess conception risk. Although concerns have been raised regarding the reliability of the assessment method we used [59; backward measure literature; but see 63], any lack of reliability should make it harder, not easier, to observe differences in behavior across the cycle. Nonetheless, future studies should seek to replicate and extend these findings using more precise hormonal assessment techniques for cycle-phase effects [47].

Another important methodological limitation pertains to statistical power vis a vis the sample size of the current study. Although we used a much larger sample size than the original study being replicated, the current study would have benefited from an even larger sample. Indeed, the current design included more experimental conditions and a more complex design than the original study being replicated, and a larger sample would have ensured even greater statistical power. Although our sample size planning was in line with Peduzzi and colleagues [50], more recent approaches suggest that the study would have benefited from an even larger sample (see Riley and colleagues [64]). Ideally the study would have included 135 participants for *each* analysis (i.e., 135 naturally cycling women as well as 135 women taking hormonal contraceptives), while in our current study, this recommended sample size was just exceeded for naturally cycling women (n = 152), yet not quite met for women taking hormonal contraceptives (n = 129). Thus, although the current study was intended to have good statistical power, a possible lack of power remains a key limitation that should be addressed in future studies. When using G*Power regarding the 3 x 2 interaction in the model with covariates (partial eta = .031), the achieved power in our study is only 0.48. Given this small effect size, we would even need a sample size of n = 399 (and not only n = 152) naturally cycling women to be truly sure whether there is an effect different from zero (including the three covariates age, relationship status and weather) for our 3 x 2 interaction. Besides, the explained variance for the 3 x 2 interaction in the model without covariates is even lower (partial Eta2 = 0.025 vs. 0.039 with covariates included), so that instead of n = 152 (achieved sample in this study) normally cycling women, a sample size of even n = 497 would be advisable for more rigorous testing. To conclude, regarding the explained variance in our 3 x 2 ANOVA including covariates for the interaction between attractiveness and fertility, post-hoc analyses revealed that we only achieved a power of 0.48 instead of the intended 0.90. Regarding literature on the color red, effect sizes are likely to be smaller than documented by previous studies and we recommend larger sample sizes for future studies aimed at replicating effects on use of the color red.

Further studies might also test whether the null effect of relationship status in this study generalizes to different populations of participants, such as somewhat older and/or more committed participants (e.g., married women). In addition, future studies testing a potential “red effect” might also attend to whether women’s use of the color red reflects a conscious versus nonconscious sexual attraction strategy. Future research might also address whether women possibly display the color red as means of intrasexual competition. That is, women might enhance their sexual attractiveness by using red attire in order to outshine female rivals. In addition, red may not be the only color that enhances attractiveness. For instance, while red leads to attractiveness via perceived sexual receptivity, fashionableness mediates the link between black and attractiveness [15]. It may be interesting to evaluate when and how different situations lead people to display red versus black as a means of attraction (e.g., in short-term versus long-term mating contexts). Moreover, it remains to be addressed whether red makeup and the use of red in women’s wardrobe might differ in their effect size. These questions provide interesting possibilities for further investigation. Yet, more efforts of replications and rigorous testing of any potential effects of color appear to be essential for drawing any conclusions regarding any influence of color on people’s choices and behavior.

Further work would also benefit from examining moderators of the “red-romance effect.” For example, the “red effect” may be more likely to emerge in short-term mating contexts than long-term contexts [15, 23, 65]. Consequently, if there is any effect, the use of red may be more closely linked with sexual desire than with long-term romantic interest. The object of attraction is likely to matter, as well. Although we manipulated the attractiveness of an ostensible partner (male research assistant), other characteristics of potential partners might affect women’s display of red. For example, signs of social dominance are often desired by women [66], maybe especially those in the fertile window [e.g., 38]. Thus, future work would benefit from examining whether women display red as a means of attractive highly dominant partners, as well as those possessing other desirable features.

There may also be instances in which woman refrain from displaying the color red for fear of evoking hostile feelings from intrasexual rivals or to avoid too much attention at occasions where social norms prescribe clothing in rather restrained colors (e.g., in some professional settings). For example, some evidence suggests that highly attractive women elicit negative judgments from female evaluators, even in domains that have little to do with mating [67–71]. Thus, women might refrain from displaying the attractiveness-enhancing color red as a way of avoiding unnecessary levels of female intrasexual competition and envy.

Finally, due to our sample of predominantly European Caucasian women, our findings may not generalize to people of other racial or ethnic backgrounds. Accordingly, future studies could test the generalizability of conditions promoting or discouraging women to wear red by including other-ethnicity participants and stimulus persons in their study design, while attending to the potentially different meanings of the color red in other cultures.

### Conclusions

Color plays a seemingly subtle, but also potentially vital, role in human social interaction. While the color red has been hypothesized to influence mating-related processes, the literature has been questioned on a variety of methodological grounds. The current study provides a methodologically rigorous conceptual replication designed to further investigate claims about factors that influence women’s use of the color red. Although women (taking hormonal contraceptives) were most inclined to display the color red when expecting to interact with a physically attractive man, there were no significant differences in women’s display of red regarding their phase of fertility in the menstrual cycle. The current study contributes to a growing state of differentiated knowledge pointing to possibly important effects of color on social perception and behavior, in that it supported prior findings that an expected social interaction partner’s attractiveness may have an effect on women’s choice of outfit, while it failed to confirm previously published effects of women’s current state of fertility on their way to dress. More broadly, the current results underline the importance of the aim to find out more about the role of evolved mating motives in human social decision-making.

## Supporting information

S1 File Supplemental materials. Supplement Results (*n* = 285) of the manuscript entitled “Antecedents of the red-romance effect: Men’s attractiveness and women’s fertility” (PONE-D-22-21067).
(DOCX)
