# Supplementary material for: Antecedents of the red-romance effect: Men’s attractiveness and women’s fertility
Source: PLoS One. 2023 Apr 11;18(4):e0284035. doi: 10.1371/journal.pone.0284035 (PMC10089354; doi:10.1371/journal.pone.0284035)
Supplement: S1 File — Supplement Results (n = 285) of the manuscript entitled “Antecedents of the red-romance effect: Men’s attractiveness and women’s fertility” (PONE-D-22-21067). (DOCX) [file pone.0284035.s001.docx]

**SUPPLEMENTAL MATERIALS**

**Supplement Results (*n* = 285) of the manuscript entitled “Antecedents of the red-romance effect: Men’s attractiveness and women’s fertility” (PONE-D-22-21067)**

Descriptive data are provided below in Table S1.

**Table S1.** Red display of all women (i.e., including other-ethnicity participants) in the attractive and the less attractive experimenter and control condition (i.e., no attractiveness information)

| **Stimulus attractiveness** | **Red not displayed** | **Red displayed** | **Total** |
| --- | --- | --- | --- |
| Attractive | 49 | 53 | 102 |
|  | 48.0% | 52.0% | 100% |
| Control | 50a | 24b | 74 |
|  | 67.6% | 32.4% | 100% |
| Less attractive | 80a | 29b | 109 |
|  | 73.4% | 26.6% | 100% |
| Total | 179 | 106 | 285 |

*Note. Significant (p < .05) between-column differences are marked by different superscripts.

We evaluated whether women’s display of the color red would be (1) affected by the attractiveness of the male experimenter and (2) associated with women’s level of fertility. Both of those predictors were included simultaneously in each model. In addition, to evaluate the robustness of each model, we repeated each analysis including participant age, relationship status, and the current weather as covariates.

**Normally cycling participants**

We first focused on normally cycling women. When analyzing the dichotomous measure (wore red or not), as can be seen in Fig 1 (left panel), 73.7% of the participants in the high-fertile phase of the cycle expecting an attractive research assistant wore red compared to 39.5% participants in the low-fertile phase of the cycle awaiting the attractive research assistant, *Chi^2^*(1) = 5.93, *p* = .015, *n* = 57), proportion difference = 34.21%, 95% CI = [0.09, 0.60]. Comparable effects could not be identified for women who were in the control condition (who did not expect an attractive or less attractive research assistant), *Chi^2^*(1) = 0.04, *p* = .84, *n* = 43), proportion difference = 3.64%, 95% C.I. = [-0.31, 0.38] or the unattractive research assistant, *Chi^2^*(1) = 0.13, *p* = .71, *n* = 55), proportion difference = 5.42%, 95% C.I. = [-0.2443, 0.3527]. We further tested this effect with a logistic regression analysis predicting the odds of wearing red from fertility (low-fertile vs. high-fertile), attractiveness of the research assistant, and age, relationship status, and weather as covariates. This analysis showed no significant effects (see Table S1).

**Fig S1.** The effects of the physical attractiveness of the male research assistant and female participants’ “fertility” in normally ovulating women versus women taking hormonal contraceptives





**Table S1.** Regressing the likelihood of wearing red (0: no vs 1: yes) from the attractiveness of the research assistant, fertility, attractiveness x fertility interaction, controlling for age, relationship status and weather for normally cycling participants

|  |  |  |  |  |  | Wald Test | | |
| --- | --- | --- | --- | --- | --- | --- | --- | --- |
| Predictor | Estimate | Robust SE | OR | 95% CI OR | z | Wald Statistic | df | p |
| Attractive RA (vs control) (AR) | 0.10 | 0.49 | 1.10 | [0.42, 2.90] | 0.20 | 0.04 | 1 | .84 |
| Unattractive RA (vs control) (UR) | -0.34 | 0.50 | 0.71 | [0.27, 1.91] | -0.68 | 0.46 | 1 | .50 |
| Fertility (F) | 0.11 | 0.75 | 1.11 | [0.25, 4.88] | 0.14 | 0.02 | 1 | .89 |
| AR x F | 1.37 | 1.01 | 3.92 | [0.55, 27.84] | 1.36 | 1.49 | 1 | .24 |
| UR x F | -0.03 | 1.04 | 0.97 | [0.13, 7.36] | -0.03 | 0.001 | 1 | .97 |
| Age | -0.02 | 0.04 | 0.98 | [0.90, 1.07] | -0.53 | 0.35 | 1 | .60 |
| Relationship status | -0.07 | 0.41 | 0.94 | [0.42, 2.11] | -0.16 | 0.03 | 1 | .87 |
| Weather | 0.56 | 0.48 | 1.76 | [0.68, 4.51] | 1.17 | 1.49 | 1 | .24 |

Notes. *N* = 155 normally cycling participants; SE = Standard error, OR = Odds Ratio, CI = Confidence Interval, RA = Research assistant, Attractive RA (AR) was coded as attractive RA (1) vs control group (0), Unattractive RA (UR) was coded as unattractive RA (1) vs control group (0), Fertility (F) was coded as 0 (low) vs 1 (high) estimated fertility, Relationship status was coded as not in a committed relationship (0) vs in a committed relationship (1), Weather was coded as bad weather (0) vs good weather (1)

The continuous dependent measure, that is the composite of the two z-transformed variables “how much” and “how obvious” was red used in the attire and/or make-up, was influenced by the attractiveness of the research assistant (F(2, 149) = 4.748, *p* = .010, partial Eta2 = .060). Post-hoc simple contrast analysis revealed, that participants displayed significantly more (and more obvious) red when awaiting an attractive research assistant (M = 0.40 [95% CI 0.14, 0.66]) compared to the control condition (M = -0.20 [95% CI -0.54, 0.14], t(149) = 2.77, p = .006, d = .64. However, women awaiting the less attractive research assistant (M = -0.078 [95% CI -0.38, 0.23] did not differ in their display of red from the control condition (t(149) = 0.54, p = .59, d = .15). The “fertility” effect (F(1, 149) = 3.21, p = .08, partial Eta2 = .08), and the attractiveness x “fertility” interaction was not significant (F(2, 149) = 1.884, p = .16, partial Eta2 = .023). As the analyses use the mean scores of the two items that are z-transformed, some of the values are lower than zero. An ANCOVA including age, relationship status and weather as covariates showed, that the main effect of attractiveness (F(2, 146) = 3.84, p = .024, partial Eta2 = .046) was still significant. All other effects did not show significant effects (Fs < 2.76, p > .10) (see Table S2).

**Table S2.** The effects of the attractiveness of the research assistant, fertility, research assistant x fertility interaction, controlling for age, relationship status, and weather

| Variable | Sum of squares | df | Mean square | F | p | η²_p_ |
| --- | --- | --- | --- | --- | --- | --- |
| Attractiveness of the RA (RA) | 6.81 | 2 | 3.41 | 3.84 | .02 | 0.05 |
| Fertility (F) | 2.45 | 1 | 2.45 | 2.76 | .10 | 0.02 |
| RA x F | 3.83 | 2 | 1.92 | 2.16 | .12 | 0.03 |
| Age | 1.74 | 1 | 1.74 | 1.96 | .16 | 0.01 |
| Relationship status | 1.71 | 1 | 1.71 | 1.92 | .17 | 0.01 |
| Weather | 0.83 | 1 | 0.83 | 0.93 | .34 | 0.006 |
| Residuals | 129.63 | 146 | 0.89 |  |  |  |

Notes. *N* = 155 normally cycling participants, Fertility (F) was coded as 0 (low) vs 1 (high) estimated fertility, Relationship status was coded as not in a committed relationship (0) vs in a committed relationship (1), Weather was coded as bad weather (0) vs good weather (1)

**Hormonal contraceptive users**

The same set of analyses was conducted on women using hormonal contraceptives. As can be seen in Fig 1 (right panel), no effects of “fertility” were observed in these women. Again, we conducted a logistic regression analysis predicting the odds of wearing red from “fertility” (“low-fertile” vs. “high-fertile”), attractiveness of the research assistant, and age, relationship status, and weather as covariates. Although the odds of wearing red was 2.08 times higher in good compared to bad weather, this model did not reveal a significant effect of weather (*p* = .095) (see Table S3).

**Table S3.** Regressing the likelihood of wearing red (0: no vs 1: yes) from the attractiveness of the research assistant, fertility, attractiveness x fertility interaction, controlling for age, relationship status and weather for hormonal contraceptive users

|  |  |  |  |  |  | Wald Test | | |
| --- | --- | --- | --- | --- | --- | --- | --- | --- |
| Predictor | Estimate | Robust SE | OR | 95% CI OR | z | Wald Statistic | df | p |
| Attractive RA (vs control) (AR) | 0.70 | 0.60 | 2.01 | [0.62, 6.54] | 1.16 | 1.36 | 1 | .25 |
| Unattractive RA (vs control) (UR) | -0.55 | 0.65 | 0.58 | [0.16, 2.06] | -0.85 | 0.79 | 1 | .40 |
| “Fertility” (“F”) | -1.09 | 1.09 | 0.34 | [0.04, 2.82] | -1.01 | 0.86 | 1 | .31 |
| AR x “F” | 1.45 | 1.25 | 4.26 | [0.37, 49.04] | 1.16 | 1.16 | 1 | .25 |
| UR x “F” | 1.49 | 1.29 | 4.42 | [0.35, 55.55] | 1.15 | 1.20 | 1 | .25 |
| Age | 0.01 | 0.04 | 1.01 | [0.93, 1.10] | 0.23 | 0.05 | 1 | .82 |
| Relationship status | 0.40 | 0.47 | 1.50 | [0.60, 3.75] | 0.86 | 0.81 | 1 | .39 |
| Weather | 0.73 | 0.43 | 2.08 | [0.90, 4.79] | 1.72 | 3.08 | 1 | .09 |

Notes. *N* = 130 hormonal contraceptive users; SE = Standard error, OR = Odds Ratio, CI = Confidence Interval, RA = Research assistant, Attractive RA (AR) was coded as attractive RA (1) vs control group (0), Unattractive RA (UR) was coded as unattractive RA (1) vs control group (0), “Fertility” (F) was coded as 0 (low) vs 1 (high) estimated fertility similar to the normally cycling group, Relationship status was coded as not in a committed relationship (0) vs in a committed relationship (1), Weather was coded as bad weather (0) vs good weather (1)

However, the attractiveness of the research assistant significantly affected the continuous composite variable (i.e., quantity and obviousness) of red women displayed, *F*(2, 124) = 4.92, *p* = .009, partial Eta2 = .074). Post-hoc tests showed that women using hormonal contraceptives display significantly more red when awaiting an attractive research assistant compared to the control condition (t(124) = 2.70, p = .008, *d* = .64), and, significant more red compared to the lower attractiveness condition (t(124) = 2.65, *p* = .009, *d* = .54). No significant differences were found between the control and the less attractive research assistant condition (t(124) = 0.52, p = .60. No further significant effects were found in the main analysis (*F*s < 1). An ANCOVA including age, relationship status and weather as covariates showed that the main effect of attractiveness is still significant in this model, F(2, 121) = 3.79, *p* = .03). Weather is no significant covariate in this model, F(1, 121) = 3.61, *p* = .060). Mated participants did not wear quantitatively more and more obvious red than single participants (F(1, 121) = 2.97, p = .09). There were no further significant effects (*Fs* < 1.15, *p* > .253) (see Table S4).

**Table S4.** The effects of the attractiveness of the research assistant, “fertility”, research assistant x “fertility” interaction, controlling for age, relationship status, and weather

| Variable | Sum of squares | df | Mean square | F | p | η²_p_ |
| --- | --- | --- | --- | --- | --- | --- |
| Attractiveness of the RA (RA) | 6.58 | 2 | 3.29 | 3.79 | .03 | 0.06 |
| Fertility (“F”) | 0.14 | 1 | 0.14 | 0.16 | .69 | 0.001 |
| RA x “F” | 0.76 | 2 | 0.38 | 0.44 | .65 | 0.007 |
| Age | 1.15 | 1 | 1.15 | 1.32 | .25 | 0.01 |
| Relationship status | 2.58 | 1 | 2.58 | 2.97 | .09 | 0.02 |
| Weather | 3.13 | 1 | 3.13 | 3.61 | .06 | 0.03 |
| Residuals | 105.04 | 121 | 0.87 |  |  |  |

Notes. *N* = 130 hormonal contraceptive users; “Fertility (F)” was coded as 0 (low) vs 1 (high) estimated fertility similar to normally cycling participants, Relationship status was coded as not in a committed relationship (0) vs in a committed relationship (1), Weather was coded as bad weather (0) vs good weather (1)
